# High KIF18A expression correlates with unfavorable prognosis in primary hepatocellular carcinoma

**DOI:** 10.18632/oncotarget.2082

**Published:** 2014-06-08

**Authors:** Weijia Liao, Guojin Huang, Yan Liao, Jianjun Yang, Qian Chen, ShengJun Xiao, Junfei Jin, Songqing He, Changming Wang

**Affiliations:** ^1^ Laboratory of Hepatobiliary and Pancreatic Surgery, Affiliated Hospital of Guilin Medical University, Guilin, Guangxi, People's Republic of China; ^2^ Guangxi Key Laboratory of Molecular Medicine in Liver Injury and Repair, Guilin, Guangxi, People's Republic of China; ^3^ Disease Prevention and Control Center of Guilin,Guilin, Guangxi, People's Republic of China; ^4^ Division of Pathology, Affiliated Hospital of Guilin Medical University, Guilin, Guangxi, People's Republic of China; ^5^ Division of Respiratory Diseases, Affiliated Hospital of Guilin Medical University, Guilin, Guangxi, People's Republic of China

**Keywords:** Hepatocellular carcinoma, KIF18A, Prognosis, Biomarker

## Abstract

This study aimed to investigate KIF18A expression in hepatocellular carcinoma (HCC) and to determine the possibility of KIF18A expression being a biomarker in HCC diagnosis or being an independent predictor of disease-free survival (DFS) and overall survival (OS) in HCC patients underwent surgical resection. KIF18AmRNA was detected in 216 cases of HCC tissues by quantitative real-time PCR (qRT-PCR) and in 20 cases of HCC tissues by semi-quantitative RT-PCR. KIF18A protein was determined in 32 cases of HCC tissues by immunohistochemistry (IHC). The survival probability was analyzed by Kaplan-Meier method, and survival curves between groups were obtained by using the log-rank test. Independent predictors associated with DFS were analyzed with Stepwise Cox proportional hazard models. High KIF18A mRNA level was detected in 154 out of 216 (71.3%) cases of HCC. The positive rate of KIF18A expression was significantly higher in liver cancer tissues than that in adjacent normal liver tissues (ANLT) from HCC patients [65.6% (21 of 32) vs. 25.0% (8 of 32), *P*=0.001]. The KIF18A expression level had positive relevance to the alpha-fetoprotein (AFP) (≥200 ng/ml), tumor size (≥5cm), clinical tumor-node-metastasis (TNM) stage and portal vein tumor thrombus (PVTT) in HCC (all *P* <0.05). A survival analysis indicated that HCC patients with higher KIF18A expression had a significantly shorter DFS and OS after resection. A multivariate analysis suggested that KIF18A upregualtion was an independent factor for DFS [hazard risk (HR)=1.602; 95% confidence interval (*CI*), 1.029-2.579; *P*=0.031] and OS (HR=1.682; 95% *CI*, 1.089-2.600; *P*=0.019). KIF18A might be a biomarker for HCC diagnosis and an independent predictor of DFS and OS after surgical resection.

## INTRODUCTION

Hepatocellular carcinoma is one of the most prevalent cancers that frequently lead to cancer-related death all over the world [1]. Despite the considerable progress in HCC diagnosis and treatment, surgical resection is still one of the most efficient treatments. During last several decades, the postoperative survival rate of HCC patients has been improved. However, due to the versatile causes of HCC, the prognosis of HCC is still not satisfactory, which is demonstrated by the low recurrence-free survival (RFS) rate (31-69%) in HCC patients within 5 years following surgical resection [2, 3]. To improve the prognosis in HCC patients, seeking more effective biomarkers in diagnosis of HCC at very early stage is very important.

Microtubule (MT) kinesin motor proteins produce force and movement by using adenosine tri-phosphate (ATP) to orchestrate various cellular processes, including mitosis, motility and organelle transportation [4]. KIF18A is a member of the kinesin superfamily whose function is not well understood. A recent report showed that it regulates chromosome congregation and suppresses kinetochore movements to control mitotic chromosome alignment in the pre-anaphase state of the mammalian cell cycle [5]. KIF18A depolymerizes microtubules during cellular division to attenuate chromosome oscillation magnitudes, and therefore promotes chromosome congregation. In addition, KIF18A regulates the stability of microtubule plus ends in the mitotic spindle. Dysfunction of KIF18A may influence chromosome segregation and/or lead to chromosome instability [6-9].

Several reports recently showed that KIF18A is involved in breast, colorectal cancer and cholangiocarcinoma [10-12]. However, to our knowledge, there is no information regarding its role in HCC progression and the potential clinical implication of KIF18A expression in HCC patients. Here, we investigated KIF18A expression in HCC and analyzed the clinical relevance of KIF18A expression to patients' clinical pathological data, and to prognosis of HCC patients after surgical resection. In this study, our findings demonstrated that KIF18A is upregulated in HCC, and KIF18A expression might be a potential biomarker for HCC histological diagnosis as well as an independent predictor of disease-free survival (DFS) and overall survival (OS) in HCC patients with surgical resection.

## RESULTS

### KIF18A expression in HCC

**Figure 1:**
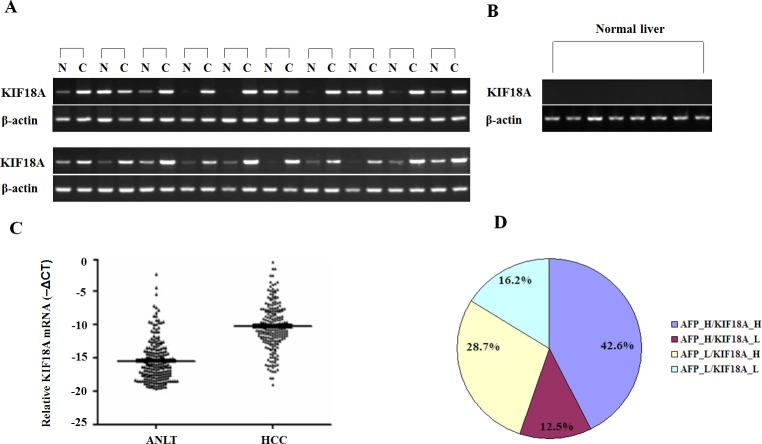
Expression pattern of KIF18A in HCC specimens and normal livers at mRNA levels (A) Representative results of semi-quantitative RT-PCR of KIF18A in liver cancer tissues [C] and their adjacent normal liver tissues [N] from 20 cases of HCC; (B) Results of semi-quantitative RT-PCR of KIF18A in 8 normal liver tissues, where β-actin was employed as an internal control. RT-PCR was generally performed in 35 thermal cycles and PCR products were visualized after electrophoresis through 2% agarose. (C) Real-time PCR analysis of KIF18A was carried out on 216 paired HCC cancer tissues and adjacent normal liver tissues (ANLT). For each sample, the relative mRNA level of KIF18A was normalized based on that of β-actin. The data shown are the Mean -ΔCT. The KIF18A mRNA expression in HCC cancer tissues was significantly higher than that in ANLT (*P* < 0.001). (D) The distribution of both KIF18A mRNA expression (the cut-off is 2^−ΔΔCt^=1) and serum AFP (the cut-off is AFP =200 ng/ml) in 216 HCC patients; the numbers in the pie indicated the percentages of KIF18A and/or AFP whose level is higher (H) or lower (L) than the cut-off value.

**Figure 2:**
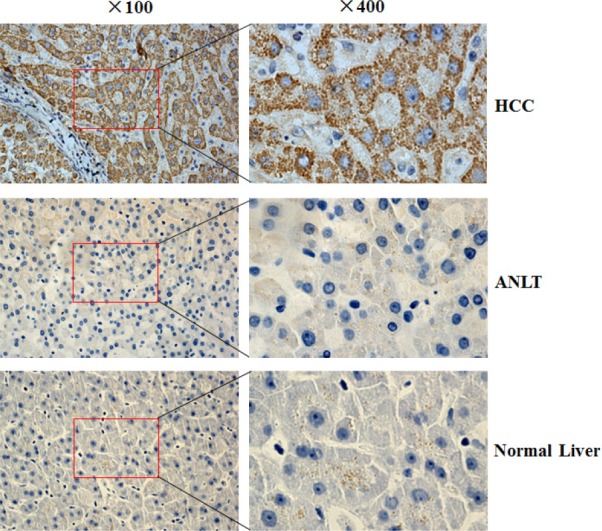
The KIF18A protein expression in HCC was determined by IHC The KIF18A protein was determined by IHC using a KIF18A antibody, the nuclei were counterstained with hematoxylin. HCC: liver cancer tissue from HCC patient; ANLT: adjacent normal liver tissue from the same HCC patient; Normal liver tissue: surrounding liver tissue from hepatic hemangioma. Original magnification: ×100 (left); ×400 (right).

Semi-quantitative RT-PCR results showed that KIF18A was significantly up-regulated in liver cancer tissues compared with ANLT in 16 of 20 (80%) HCC patients (Fig. 1A), while its expression in 8 cases of normal liver tissues from hepatic hemangioma' surrounding liver tissues was undetectable (Fig. 1B). To confirm RT-PCR results, Real-time PCR analysis was performed to detect KIF18A expression in 216 cases of HCC specimens. Compared with ANLT, KIF18A expression was significantly increased (2^−ΔΔCt^≥1) in 154 cases (71.3%), but decreased (2^−ΔΔCt^<1) in 62 cases (28.7%) (*P*<0.001, Fig. 1C). Interestingly, we found that the increase of KIF18A mRNA expression in HCC tissues (2^−ΔΔCt^≥1) and the increase of AFP (≥200 ng/ml) in sera from HCC patients were not happened concurrently. Both of them increased in 92 cases (42.6%), AFP alone increased in 27 cases (12.5%) and KIF18A expression alone increased in 62 cases (28.7%) (Fig. 1D). These results indicated that KIF18A might be a candidate of novel biomarker for HCC histology diagnosis. If combining serum AFP and tissue KIF18A expression, HCC diagnosis rate reached more than 80% in our study. We further conducted immunohistochemical assay to detect KIF18A protein in 32 cases of HCC tissues, and found that 21 of 32 (65.6%) cancer tissues had KIF18A positive staining while only 8 of 32 (25.0%) ANLT and none of 8 normal liver tissues from hepatic hemangioma had KIF18A positive staining (Fig. 2).

### Correlation between KIF18A expression level in HCC tissues and the clinical pathological data

After obtained KIF18A expression results, we performed statistical analysis to determine the relevance between KIF18A and clinical pathological data (Table 1), and found that KIF18A expression was correlated with serum AFP level (≥200 ng/ml) (χ^2^ = 4.684, *P* = 0.030), tumor size (≥5cm) (χ^2^ = 6.787, *P* = 0.009), clinical TNM stage (χ^2^ = 14.312, *P* <0.001) and PVTT (χ^2^ = 7.228, *P* = 0.007), but was not obviously related to age, gender, family history, HBsAg, liver cirrhosis, distant metastasis, or postoperative recurrence (all *P* >0.05).

**Table 1:** Correlation between the clinicopathologic variables and KIF18A mRNA expression in HCC

Clinical character	variable	No.of patients	KIF18A mRNA		χ^2^	*p* value
			Low n (%)	High n (%)		
Age (years)	<55	142	42 (29.6)	100 (70.4)	0.155	0.694
	≥55	74	20 (27.0)	54 (73.0)		
Gender	Male	185	49 (26.5)	136 (73.5)	3.097	0.078
	Female	31	13 (41.9)	18 (58.1)		
Family history	No	183	50 (27.3)	133 (72.7)	1.117	0.291
	Yes	33	12 (36.4)	21 (63.6)		
HBsAg	Negative	38	12 (31.6)	26 (68.4)	0.186	0.666
	Positive	178	50 (28.1)	128 (71.9)		
AFP (ng/mL)	<200	97	35 (36.1)	62 (63.9)	4.684	0.030
	≥200	119	27 (22.7)	92 (77.3)		
Median size (cm)	<5	54	23 (42.6)	31 (57.4)	6.787	0.009
	≥5	462	39 (24.1)	123 (75.9)		
Cirrhosis	No	20	4 (20.0)	16 (80.0)	0.816	0.366
	Yes	196	58 (29.6)	138 (70.4)		
Tumor number	Single	146	43 (29.5)	103 (70.5)	0.123	0.725
	Multiple	70	19 (27.1)	51 (72.9)		
TNM stage	I-II	106	43 (40.6)	63 (59.4)	14.312	<0.001
	III-IV	110	19 (17.3)	91 (82.7)		
PVTT	No	161	54 (33.5)	107 (66.5)	7.228	0.007
	Yes	55	8 (14.5)	47 (85.5)		
Distant metastasis	No	197	58 (29.4)	139 (70.6)	0.596	0.440
	Yes	19	4 (21.1)	15 (78.9)		
Recurrence	No	148	46 (31.1)	102 (68.9)	1.298	0.255
	Yes	68	16 (23.5)	52 (76.5)		

HBsAg, hepatitis B surface antigen; AFP, alpha-fetoprotein; TNM, tumor-node-metastasis; PVTT, portal vein tumor thrombus; N, number of patients.

### Relevance among KIF18A mRNA, clinical pathological index and postoperative DFS or OS

**Figure 3:**
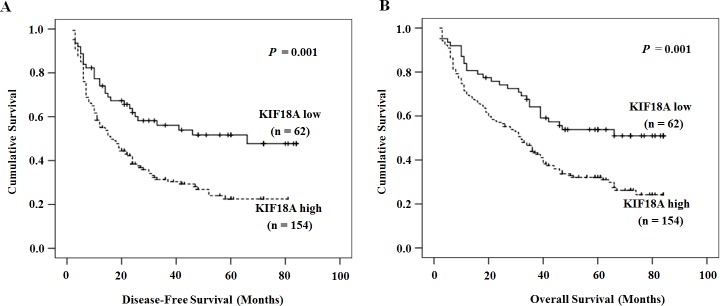
The relationship between KIF18A expression and DFS or OS Patients with high KIF18A expression had a shorter DFS (A) and OS (B). The solid line represents the patient with low KIF18A expression, whereas the dashed line represents the patients with high KIF18A expression.

Kaplan-Meier survival analysis showed that a higher KIF18A expression was associated with a shorter DFS and OS (Fig. 3). Univariate analysis revealed obvious association of clinical parameters with both DFS and OS (Table 2). Mean DFS in patients with high KIF18A expression was 30.84 months [>95% confidence interval (*CI*), 25.82-35.86] compared with 50.14 months (95% *CI*, 41.14-59.15) in patients with low KIF18A expression (*P =*0.001). Mean OS in high KIF18A expression group and low KIF18A expression group was 39.26 months (34.33-44.19) and 55.06 months (47.03-63.10), respectively (*P =*0.001). Besides high KIF18A expression, size of tumor ≥5cm, multiple tumor number, III-IV of TNM stage, the AFP value (≥200 ng/ml), recurrence together with PVTT and distant metastasis were associated with a shorter DFS and OS. (Table 2).

**Table 2:** Association between KIF18A expression, clinical parameters and disease-free survival/overall survival

Clinical character	Category	No.of patients	Disease-free survival (months)			Overall survival (months)		
			Mean	95% CI	*p* value	Mean	95% CI	*p* value
KIF18A expression	Low	62	50.14	41.14-59.15	0.001	55.06	47.03-63.10	0.001
	High	154	30.84	25.82-35.86		39.26	34.33-44.19	
Age (years)	<55	142	36.97	31.09-42.84	0.846	43.16	37.75-48.58	0.790
	≥55	74	37.06	29.41-44.71		45.21	38.10-52.32	
Gender	Female	31	39.84	28.58-51.11	0.171	53.49	42.34-64.63	0.108
	Male	185	35.73	30.76-40.69		42.21	37.57-46.86	
Family history	No	183	35.72	30.68-41.66	0.226	42.43	37.78-47.08	0.165
	Yes	33	44.40	32.28-56.53		51.02	39.90-62.13	
HBsAg	Negative	38	35.28	24.04-46.53	0.938	45.42	35.63-55.22	0.870
	Positive	178	37.23	32.07-42.38		43.50	38.71-48.31	
AFP (ng/mL)	<200	97	41.79	34.73-48.84	0.052	49.70	43.42-55.97	0.017
	≥200	119	32.96	26.79-39.12		38.85	33.07-44.63	
Tumor size (cm)	<5	54	62.80	54.34-71.27	<0.001	68.56	62.02-75.09	<0.001
	≥5	162	28.28	23.40-33.16		35.56	30.88-	
Cirrhosis	No	20	30.59	15.31-45.86	0.329	37.90	24.05-51.75	0.455
	Yes	196	37.43	32.52-42.35		44.37	39.85-48.90	
Tumor number	Single	146	43.20	37.40-48.99	<0.001	49.75	44.56-54.94	<0.001
	Multiple	70	23.03	16.54-29.51		31.36	24.48-38.23	
TNM stage	I-II	106	51.07	44.41-57.74	<0.001	58.19	52.63-63.75	<0.001
	III-IV	110	22.97	17.70-28.23		30.01	24.58-35.44	
PVTT	No	161	42.71	37.14-48.27	<0.001	49.66	44.69-54.63	<0.001
	Yes	55	20.06	13.47-26.64		26.83	19.91-33.76	
Distant metastasis	No	197	38.67	33.72-43.62	0.005	44.98	40.41-49.55	0.036
	Yes	19	17.73	9.21-25.65		31.52	20.26-42.78	
Recurrence	No	148				37.85	32.64-43.06	<0.001
	Yes	68				56.96	50.23-63.69	

### High KIF18A expression as an independent predictor of DFS or OS

We used Cox proportional hazard model to determine the relationship of eight factors including high KIF18A expression to DFS and OS in HCC patients with surgical resection. Among these factors, only AFP ≥200 ng/ml and recurrence were related to OS, recurrence was only used as an independent predictor factor for OS. Other six factors (size of tumor >5 cm, multiple tumor number, III-IV of TNM stage, PVTT, distant metastasis and high KIF18A expression) were analyzed with the stepwise multivariate Cox proportional hazard model for both DFS and OS. The results showed that size of tumor >5 cm (HR, 3.119; 95% *CI*, 1.795-5.421; *P* <0.001), III-IV of TNM stage (HR, 1.650; 95% *CI*, 1.076-2.529; *P*=0.022) and high KIF18A expression (HR, 1.602; 95% *CI*, 1.029-2.579; *P* =0.031) were independent predictors for DFS (Table 3). Size of tumor >5 cm (HR, 2.614; 95% *CI*, 1.495-4.568; *P*=0.001), III-IV of TNM stage (HR, 1.895; 95% *CI*, 1.235-2.907; *P*=0.003), recurrence (HR, 2.074; 95% *CI*, 1.374-3.131; *P* =0.001) and high KIF18A expression (HR, 1.682; 95% CI, 1.089-2.600; *P* =0.019) were independent predictors for OS (Table 3).

**Table 3:** Cox multivariate proportional hazard model of independent predictors on DFS and OS

Variable	Hazard ratio (95%CI)	*P* value
Disease-free survival		
Tumor size, cm (≥5 *vs* <5 )	3.119 (1.795-5.421)	<0.001
Tumor number (multiple vs single)	1.302 (0.903-1.878)	0.157
TNM stage (III-IV vs I-II)	1.650 (1.076-2.529)	0.022
PVTT (yes *vs* no)	1.372 (0.915-2.058)	0.126
Distant metastasis (yes *vs* no)	1.574 (0.915-2.711)	0.101
KIF18A expression (high *vs* low)	1.602 (1.029-2.579)	0.031
Overall survival		
AFP (ng/mL) (≥200 *vs* <200)	1.242 (0.871-1.770)	0.232
Tumor size, cm (≥5 *vs* <5 )	2.614 (1.495-4.568)	0.001
Tumor number (multiple *vs* single)	1.389 (0.962-2.006)	0.080
TNM stage (III-IV *vs* I-II)	1.895 (1.235-2.907)	0.003
PVTT (yes *vs* no)	1.272 (0.844-1.916)	0.250
Distant metastasis (yes *vs* no)	1.076 (0.624-1.857)	0.791
Recurrence (yes *vs* no)	2.074 (1.374-3.131)	0.001
KIF18A expression (high *vs* low)	1.682 (1.089-2.600)	0.019

CI, confidence interval; TNM, tumor-node-metastasis; PVTT, portal vein tumor thrombus; AFP, alpha-fetoprotein; DFS, disease-free survival; OS, overall survival.

## DISCUSSION

In our current study, we investigated KIF18A expression in HCC patients and found that KIF18A expression at both mRNA and protein level was significantly up-regulated in liver cancer tissues compared with that in ANLT, we also found a positive correlation between KIF18A expression and clinical characteristics, including AFP ≥200 ng/ml, tumor size >5 cm, TNM stage III/IV and PVTT appearance, which are closely related to a bad outcome of HCC. KIF18A expression was well correlated with adverse prognostic factors and shorter survival, suggesting that this mitotic protein might be associated to aggressive features in HCC, which is consistent with a previous report that HCC cells might take advantage of KIF18A overexpression to control mitotic chromosome alignment and promote cell division [14].

AFP levels have been widely used for diagnosis as well as surveillance of HCC. However, the sensitivity and specificity of AFP levels for HCC surveillance have some shortcomings [15], because AFP levels may be normal in up to 40% of patients with HCC, particularly during the early stages of HCC. Therefore, it is urgent to identify some factors affecting the survival of HCC patients, including conventional clinicopathological variables and novel molecular markers [16]. Our study suggested KIF18A might be a novel biomarker for HCC pathological diagnosis. If use both of AFP and KIF18A as biomarkers, the diagnostic positive ratio of HCC patients could be improved dramatically.

Univariate analysis revealed that high KIF18A expression, median size of tumor ≥5cm, multiple tumor number, III/IV of TNM stage, PVTT and distant metastasis were associated with a shorter DFS. Previous studies showed that tumor number was an important determinant for prognosis of HCC patients underwent several kinds of treatments [17]. Obviously, individuals with single HCC tumor had relatively better survival and better prognosis than those with multi-nodular tumor [16]. The main cause of metastasis and recurrence of HCC is that HCC cells tend to invade portal veins and then PVTT appears. PVTT, a unique disseminating manner of HCC, is associated with poor prognosis of HCC [18] and is identified as a special type of metastasis in HCC [19].

It is very interesting that KIF18A high expression, tumor size ≥5cm, and TNM stage III/IV were also identified as independent prognostic factors for DFS and OS by multivariate analysis. The prognostic relevance of AFP [20, 21] and tumor size [22, 23] for DFS in HCC patients was confirmed by previous studies. The tumor size>5cm was identified as a significant risk factor of recurrence after liver resection [22, 23]. Generally, small HCC tumors (median size <5cm) have a better prognosis [24, 25], However, larger tumors (>5cm) are reported to be associated with greater likelihood of vascular invasion and higher recurrences risk [22, 23, 26].

The high transferability and invasiveness of the malignant tumor are the key factors of tumor's development [27]. Therefore, a major focus of cancer research in HCC today is to find effective drugs to inhibit tumor's invasion and migration.

Based on KIF18A up-regulation in HCC tissues, it might serve as a useful therapeutic target for personalized therapy in HCC patients with high KIF18A expression. In addition, given the fact that KIF18A plays critical roles in modulating microtubule (MT) dynamics and mitosis, MTs and MT-associated proteins might also be significant drug targets for cancer chemotherapy [28, 29].

In summary, our current study suggested that KIF18A might play an important role in HCC carcinogenesis and prognosis, KIF18A might serve as a diagnostic marker, a prognostic marker as well as a therapeutic target for HCC. However, the underlying molecular mechanisms remain unclear and should be our future research direction. In addition, personalized therapy in HCC patients with high KIF18A expression at early stage can be pursued in our future work and long-term follow-up of HCC patients with high KIF18A expression is needed.

## MATERIALS AND METHODS

### The source of specimens

216 cases of tissues from HCC patients including liver cancer tissues and adjacent normal liver tissues (ANLT) were obtained from Affiliated Hospital of Guilin Medical University from 2001 to 2007. These subjects were confirmed by the clinical, serological, ultrasonography (US), Catscans (CT), magnetic resonance imaging (MRI) and pathological examination, and the diagnoses were consistent with “Primary Liver Cancer Clinical Diagnosis and Staging Criteria”. None of these patients had received transhepatic arterial embolization or chemotherapy before surgical resection. Clinicopathologic characteristics for patients were shown in Table 1. The surrounding tissues from 8 cases of hepatic hemangioma were thought as normal liver tissues and confirmed by pathological examination. Samples for gene expression assay were immediately frozen in liquid nitrogen and then stored at −80°C in a freezer until use. Samples for immunohistochemistry assay were fixed with formalin and then embedded with paraffin.

To collect prognosis data, patients underwent surgical resection received follow-up examinations, including serum alpha-fetoprotein (AFP) test and ultrasonography (US) examination every 2 months, chest radiography every 6 months during the first two postoperative years and at 3-6 month intervals thereafter. Catscans (CT) or magnetic resonance imaging (MRI) was performed if an abnormal result of AFP test or ultrasonography (US) examination was found. The mean postoperative follow-up term was 36.0 months (median, 21.0 months; range, 2.0 to 84.0 months). DFS was measured from the date of surgery to the date of recurrence, metastasis, death or last follow-up. OS was measured from the date of surgery to the date of death or last follow-up. Study protocols were approved by the Hospital Ethics Committee of Guilin Medical University, and written informed consent was obtained from patients based on the Declaration of Helsinki.

### RNA extraction and cDNA synthesis

Total RNA was isolated from HCC frozen samples by using TRIzol (Invitrogen) reagent. RNA concentration was determined by spectrophotometry, and total RNA integrity was monitored by visualization of ribosomal RNAs (28S and 18S) on 1.2% agarose gel. First strand cDNA was synthesized using PrimeScript RT reagent Kit (TaKaRa) according to manufacturer's instruction.

### Reverse transcription-PCR and quantitative real-time PCR

The KIF18A and β-actin primers were designed based on gene sequences in Gene Bank (KIF18A, NM_031217.3) using Primer Premier 5.0 software, and verified by PubMed Blast comparative analysis. The primers were synthetized by Shanghai Biological Engineering Co., Ltd (Shanghai, China). KIF18A upstream primer sequence was 5′-CAGTTCAGCCTATTCCTT-3′, its downstream primer sequence was 5′-TATCACTGTTTATGTTTGAGC-3′, and the length of the amplified fragment was 300 bp. β-actin's upstream primer sequence was 5′-TCACCCACACTGTGCCCATCTACGA-3′, its downstream primer sequence was 5′-CAGCGGAACCGCTCATTGCCAATGG-3′ and the length of the amplified fragment was 295 bp. The PCR reaction was performed in 20μl reaction system with TaKaRa PCR kit under the reaction condition as following: 94°C for 5 minutes, then 35 cycles (for KIF18A) or 25 cycles (for β-actin) of 94°C for 30s, 55°C for 30s, 72°C for 30s, followed with 5 min incubation at 70°C. Reaction was terminated at 4°C. The PCR products were analyzed on a 2% agarose gel and visualized under UV light after ethidium bromide staining.

The quantitative real-time PCR (qRT-PCR) was performed according to the instructions of SYBR Premix Ex Taq. The primers for KIF18A were: 5′-AAAAAGTGGTAGTTTGGGCTGA-3′ (sense) and 5′-CTTTCAAGGGAGATGGCATTAG-3′ (antisense). The sequences of inner control gene β-actin were 5′-GACAGGATGCAGAAGGAGATTACT-3′ (sense) and 5′-TGATCCACATCTGCTGGAAGGT-3′ (antisense). qRT-PCR amplification and data analysis were performed using the ABI Prism 7500 Sequence Detector System Applied Biosystems (Foster City, CA, USA). Each cDNA sample was mixed with 15 μl of Master mix (SYBR® Green PCR Master Mix, Applied Biosystems). qRT-PCR was performed with a protocol given below: an initial denaturation step at 95°C for 10 min, followed by 40 cycles of denaturation at 95°C for 2 sec, annealing at 55°C for 5 sec, and extension at 72°C for 15 sec. KIF18A mRNA was calculated using the delta-delta CT (ΔΔCt) method [13] and normalized to β-actin.

### Immunohistochemistry assay

Sections were dewaxed with xylene, followed by rehydration in graded alcohols. Antigen was retrieved by pressure cooking for 3 minutes in citrate buffer (pH = 6.0), and washed in phosphate buffered saline (PBS), then immersed in 3% hydrogen peroxide for 20 minutes to block endogenous peroxidase activity. Sections were pre-incubated with 10% goat serum at room temperature for 30 minutes to block nonspecific reaction. Subsequently, sections were incubated with rabbit polyclonal anti-KIF18A antibody (catalog 19245, Proteintech ^TM^ Company, 1:200 dilution) overnight in a moist chamber at 4°C, then washed with PBS, incubated with biotinylated goat anti-rabbit antibody for 1 hour at room temperature, and stained with 3, 3- diaminobenzidine tetrahydrochloride (DAB). Finally, the sections were counterstained with Mayer's hematoxylin, dehydrated, and mounted. A negative control was obtained by replacing the primary antibody with normal rabbit serum. Semi-quantitative IHC detection was used to determine the KIF18A protein level, and the stained tissue sections were evaluated by two pathologists on separate occasions using a 4-point scale as follows: positive cell counts, grades 0-3 (0, no positive cells; 1, <25% positive cells; 2, 25%-50% positive cells; 3, >50% positive cells).

### Statistical analysis

Results were analyzed with SPSS13.0 statistical software. Correlation between KIF18A expression and clinicopathologic parameters was evaluated using the Chi-square (χ^2^) test, and quantitative variables were analyzed by the independent *t* test. The survival probability was estimated by Kaplan-Meier method, and the comparison of survival curves between groups was done with the log-rank test. Independent predictors associated with DFS were analyzed with Stepwise Cox proportional hazard models. The statistical significance of the differences between mean values was determined by *P* <0.05.
